# Supplementary material for: Data Mining Identifies CCN2 and THBS1 as Biomarker Candidates for Cardiac Hypertrophy
Source: Life (Basel). 2022 May 12;12(5):726. doi: 10.3390/life12050726 (PMC9147176; doi:10.3390/life12050726)
Supplement: Supplementary file 1 [file life-12-00726-s001.zip › Supplementary material file S1.pdf]

Supplementary material

Western blot analysis method

For quantitative Western blot analysis, the following protocol was used. Samples were prepared by mixing the 3/4th volume sample with 1/4th volume NuPAGE™ LDS Sample Buffer 4X (Invitrogen) for non-reduced samples (Thrombospondin 1) with DTT added to a final concentration of 100mM for reduced samples (Nestin). Sample-mixes were heated at 95 °C for 5 min. Samples were loaded onto a NuPAGE™ 4-12% Bis-Tris Gels with 17 wells (Invitrogen, Waltham, MA, USA), using NuPAGE™ MOPS SDS Running Buffer (Invitrogen). Wells were loaded with either 15µL sample-mix per well or 2µL µL of the Precision Plus Protein Dual Color Standards (Bio-Rad laboratories, Inc.). The SDS PAGE ran at 200 V for 45 min. Proteins were transferred to an activated (30 sec methanol soak) 0.45 µm Immobilon-FL PVDF membrane (Merck Millipore, Ltd.) using TOWBIN+20% methanol as transfer buffer and Trans-Blot SD (Bio-Rad laboratories, Inc) for blotting. The transfer ran for 1 h with 140 mAmp and 24 V. After transfer the membrane was imme-diately transferred to blocking buffer (PBS pH 7.4, 4% w/v BSA, 0.05% w/v Sodium Azide) for 20 minutes. The media was then replaced with blocking buffer supplemented with 0,02% v/v Tween 20 and 1:500 primary antibody (Thrombospondin 1 #MA5-13398 Thermofisher Scientific, Nestin #MA1-110 Thermofisher Scientific). Membranes were incubated at 4°C over night. After incubation membranes were washed 5x5 min in PBSt (PBS pH 7.4, 0.02% Tween 20) and transferred to blocking buffer supplemented with 0.02% v/v Tween 20, 0.02% w/v SDS, 0.05% w/v Sodium Azide and 1:20000 secondary antibody (IRDye® 680RD Donkey anti-Mouse IgG, Licor Biosciences). Membranes were incubated with secondary antibody for 1h at RT after which they were washed with PBSt. Membranes were imaged with an Oddyssey CLx (Li-Cor, Inc) and the band strength was assessed using Image Studio Lite (Li-Cor, Inc).
