# Supplementary figures and images for: Data Mining Identifies CCN2 and THBS1 as Biomarker Candidates for Cardiac Hypertrophy
Source: Life (Basel). 2022 May 12;12(5):726. doi: 10.3390/life12050726 (PMC9147176; doi:10.3390/life12050726)

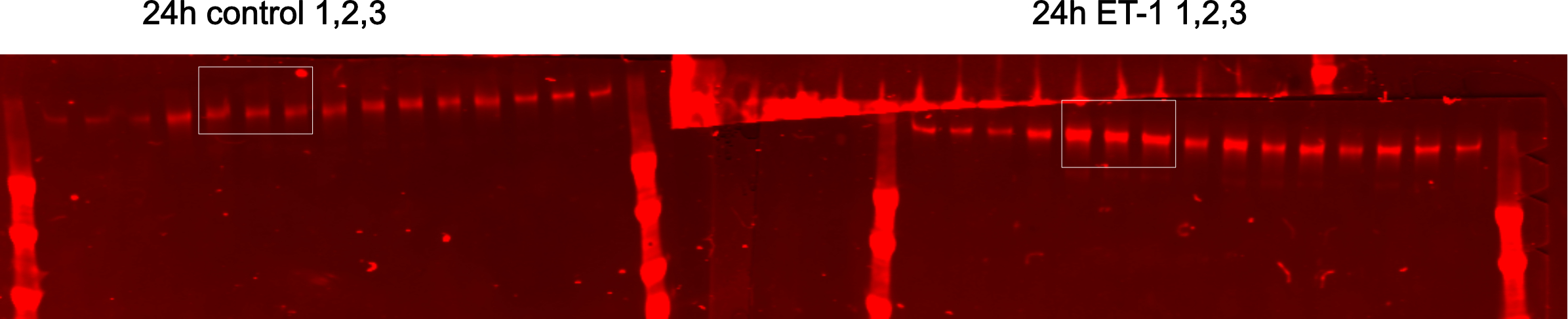

Supplement: Supplementary file 1 [file life-12-00726-s001.zip › Figure S1.jpg]
